# Supplementary honey bee (*Apis mellifera* L.) pollination enhances fruit growth rate and fruit yield in *Paeonia ostii* (family: Paeoniaceae)

**DOI:** 10.1371/journal.pone.0272921

**Published:** 2022-08-25

**Authors:** Kaiyue Zhang, Yuying Li, Kaili Sun, Junyi Bao, Chunling He, Xiaogai Hou

**Affiliations:** 1 College of Agriculture / Tree Peony, Henan University of Science & Technology, Luoyang, China; 2 College of Horticulture and Plant Protection, Henan University of Science & Technology, Luoyang, China; Gomal University, PAKISTAN

## Abstract

Insufficient pollination leads to low and unstable production of oil tree peony. Supplementary managed honeybees (*Apis mellifera* L.) in agricultural ecosystems is a common practice for addressing the problem. At this study site (N 34°38′30″ and E 112°39′43″, with an altitude of 125.5 m), we set up four pollination areas (low-density bee pollination group (LDBP), high-density bee pollination group (HDBP), blank control group (CK1) and field control group (CK2)) to examine the pollination effectiveness of different densities of honeybee supplementation on oil tree peony (*Paeonia ostii*). Our work demonstrated that bee-pollination increased fruit size and growth rate. On average, bee-pollinated (LDBP) plants produced 63.16% more number of seeds per plant, showed also 53.47% more weight of seeds per plant than those in CK2. Also, seeds of LDBP contained, on average, 26.15% more oil content than CK2. Kernel percent and seed oil fatty acid content, however, were unaffected (F = 1.759, p = 0.074). Compared with LDBP, weight of seeds per plant and oil content with HDBP decreased by 21.89% and 2.63%, respectively. Following the same trend, compared with LDBP, HDBP slowed fruit growth and reduced fruit size. Our results showed that insufficient pollination limits fruit set in oil tree peony, while supplementary reasonable bee density in the field for pollination is an important strategy to maximize fruit yield.

## 1. Introduction

More than 70% of all agricultural crops depend to varying degrees on pollinators to obtain sufficient pollen to maximize ovule fertilization, thereby maximize fruit or seed production and quality [1, 2]. As the increasing planting acreage of flowering crops, the demand for pollination services also increases [3]. However, with the loss and fragmentation of habitats, intensification of agriculture, agricultural and industrial chemicals, parasites and diseases, and the introduction of alien species, pollination services level in agroecosystems also dropped [4–8]. Pollination shortage is an important factor in crop yield, especially for crops those highly dependent on pollinators. In order to maximize the shortage of supplementary pollination, flowering crop pollination needs to be unified and stable in time and space [9, 10]. This spatiotemporal stability is usually achieved through the pollination service provided by wild bees [10] and the use of a few managed pollinators. In agroecosystems, the use of wild bees and providing managed pollinators, like honeybees (*Apis mellifera*), at times of high demand, are the main strategies to stabilize pollination levels and mitigate pollination deficits [11, 12].

*A*. *mellifera* is the most common managed crop pollinator worldwide [13], either in open or enclosed pollination systems [14]. As an important pollinating colony, the value of *A*. *mellifera* for crop pollination in North America, is €13.82($14.8 billion) annually. This figure is undoubtedly growing year by year [15]. Emerging research on honeybees also demonstrates their effectiveness as pollinator for common oil-bearing crop [16–20]. Moreover, bee pollination can also increase fruit size, growth rate, shorten the ripening period, and improve the sugar content, vitamin levels, taste, color, and fruit appearance [21–25].

To maximize crop productivity, large numbers of colonies are provided in the field at the onset of flowering of the target crop [26]. Currently, there is no consensus on the optimal colony density to maximize crop yield, and recommendations are highly variable, even within the same crops and cultivars [27, 28]. There is little application of bee pollination management strategy in the production of oil tree peony. It is very necessary to continue to practice.

Oil tree peony is an emerging woody oil crop with high oil value and economic value. Importantly, it is an excellent woody oil crop (Oil content, 20~35% and UFAs, >90%) relatively to other oil crops [29–31]. Tree peony is a bisexual flower line, with typical entomophilous flower characteristics: the flower is large, white, fragrant, with copious amounts of pollen and bright yellow [32, 33]. Apomixis is absent in tree peony, there is no automatic self-pollinated phenomenon, with the strains of cross-pollination to produce a small amount of seeds, with weak self-nature (only 0.4 seeds/fruit) [34, 35]. Inadequate pollination has been suggested as an important factor in the limiting of the oil tree peony yield. Pollen-carrying vectors play a key role in the pollination of oil tree peony because a considerable number of fruits are produced only through cross pollination [36–38]. Oil tree peony flowers are visited by a variety of insects including bees, ants, beetles and flies, among them, the dominant flower-visiting insects are *A*. *mellifera*, *A*. *c*. *cerana*, and bumble bees [36–38].

Although honey bees have been described as efficient pollinators, the lack of nectar reward in oil tree peony flowers makes them poorly attractive to honey bees. For this reason, we set up a pollinator control net room supplemented with bees to evaluate pollination deficit in oil tree peony field. Previous studies have shown that *A*. *mellifera* are more efficient pollinators than *Bombus terrestris* on oil tree peony, and that pollination by *A*. *mellifera* can accelerate fruit development rates and yield [37, 38]. This study compared the effects of different pollinator colony densities on the fruit development rate and fruit yield of oil tree peony, and provided theoretical support for further utilization of pollinator bee resources in oil tree peony field and high yield and high benefit of oil tree peony.

## 2. Materials and methods

### 2.1. Sampling area

The study was conducted in an oil tree peony plantation in the East Garden of Yibin District, Luoyang city, China (N 34°38′30″ and E 112°39′43″, with an altitude of 125.5 m) from April to August 2019. The oil tree peony planted in this location is *Paeonia ostii* T. Hong et J.X. Zhang. The planting area was 2.40 ha. Plants age at least nine or more years old, which has entered the high-yield period.

### 2.2. Experimental design

Building net rooms were set in parallel at east-west direction of the central location of the field. Each net room was 45 m length and 8 m width with a side wall of 2.1 m height and a roof ridge of 3.2 m height. A nylon polyethylene mesh (2 mm) was used to construct the walls and ceilings of the pollination rooms.

### 2.3. Pollination treatments

**Fig 1:**
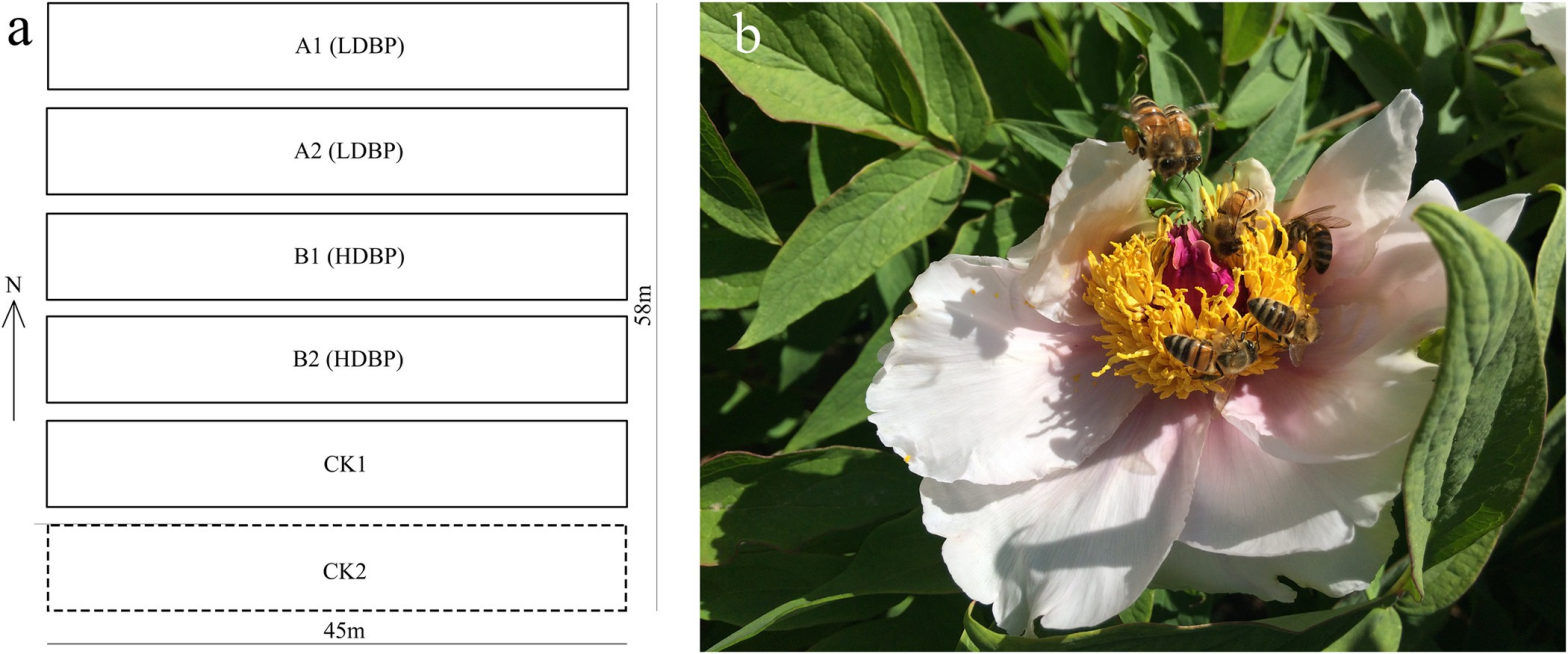
Plan graph of the net pollination rooms built in the oil tree peony plantation.

A total of five pollinating net rooms were built, of which two net rooms were placed one box of bees (approx. 8000 workers per hive, 22 bees/m2), two net rooms were placed two boxes of bees (approx. 8000 workers per hive, 44 bees/m2), and one net room was a blank control net room without pollinators. A field natural control area was set parallel to the blank net room (**Fig 1**). All oil tree peony plants in the test field, select the same plant age, and use the same standardized scientific management.

Group (A1/A2): One beehive was placed in a net room. This room was designed as low-density bee pollination group (LDBP).

Group (B1/B2): Two beehives were placed in a net room. This room was designed as high-density bee pollination group (HDBP).

Group (C): Was the blank control group (CK1) in which bees were excluded, while wind and self- pollinations were allowed.

Group (D): Was the field control group (CK2) where pollinators, wind and self-pollination were permitted.

Same management practices typically applied in each of the experimental plots of oil tree peony. The experimental race of the bee used is *Apis mellifera* L. The bees were supplied by Luoyang Lilou Apiary. The type of the utilized bee-hive is Langstroth’s wooden hive (Lid size: 55cm×45cm×8cm; Box ring size: 51cm×41cm×26cm).

### 2.4. Fruit growth parameters

The fruit of oil tree peony in Luoyang often develops from April to August and goes through four developmental stages. In April 12^th^ (early spring season of 2019), each area was marked with 10 buds at the same developmental stage, for a total of 60 buds. As previously indicated, treatments (plots) were designated as LDBP, HDBP, CK1, and CK2. Referring to previous research [39], determination of fruit development parameters begins at the end of the flowering period, vernier calipers were used to measure the diameter of the developing tree peony fruits and other measurements, i.e. follicle diameter and length, fruit diameter, and seed diameter [40]. Measurements were done every 10 days, until harvest.

### 2.5. Yield parameters and oil quality

In each area, the marked and the remaining unmarked random 80–100 tree peony intact fruits were harvested after ripening. The following yield parameters were measured and calculated per plant: number of seeds per fruit, weight of seeds per fruit, number of seeds per plant and weight of seeds per plant (measured by FA1004 electronic balance in Laboratory of Peony Physiology and Ecology, Henan University of Science and Technology; Max: 100g, d = 0.1mg, e = 10d). Kernel percent (total kernel weight / total seeds weight). Oil content (oil weight / kernel weight), which was extracted using a HA220-50-06 supercritical extraction device (extract Ⅱ: 40°C, 30MPa; separation Ⅰ: 40°C, 10MPa; separation Ⅱ: 35°C, 5MPa; extraction agent: CO_2_; materials: peony seed kernels). Oil quality, represented as the content of fatty acids in the seed oil was measured using A 5977B GC/MSD gas chromatography-mass spectrometer (sample size: 0.2g) [41].

### 2.6. Data analysis

Excel 2016, Origin 18.0, and SPSS 20.0 were used to conduct statistical analyses of the data. One-way ANOVAs were performed in SPSS 20.0 to test the impact of bee pollination on the rate of fruit growth and development, yield indicators, and the oil quality. Subsequently, a Duncan’s new complex range test was used to determine significant differences (at p<0.05 and p<0.01) between treatment means.

## 3. Results

### 3.1. Fruit development

**Fig 2:**
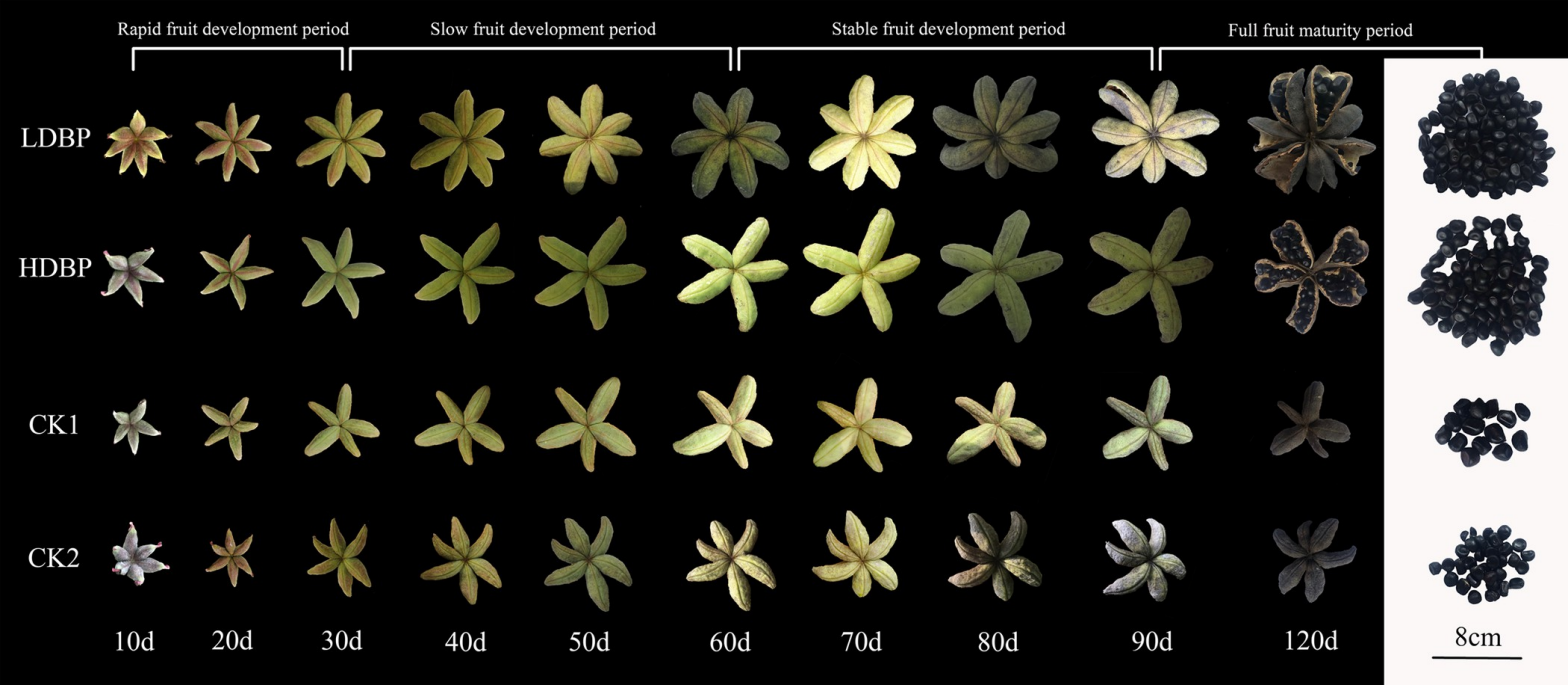
Dynamic changes in fruit development of oil tree peony.

The fruit is composed of aggregate follicles. The fruit gradually enlarges during development, changing from green (with red fluff) to yellowish green (with brownish yellow fluff) to crab yellow (harvesting) to gradually black (post-ripening). There was no difference in fruit maturity among the four treatments, but compared with the control group, the fruit of the bee-pollination group was more likely to crack naturally at the ripening stage and the grain was more likely to fall off (**Fig 2**).

### 3.2. Fruit growth rate

#### 3.2.1. Accumulative increase rate of fruit

**Fig 3:**
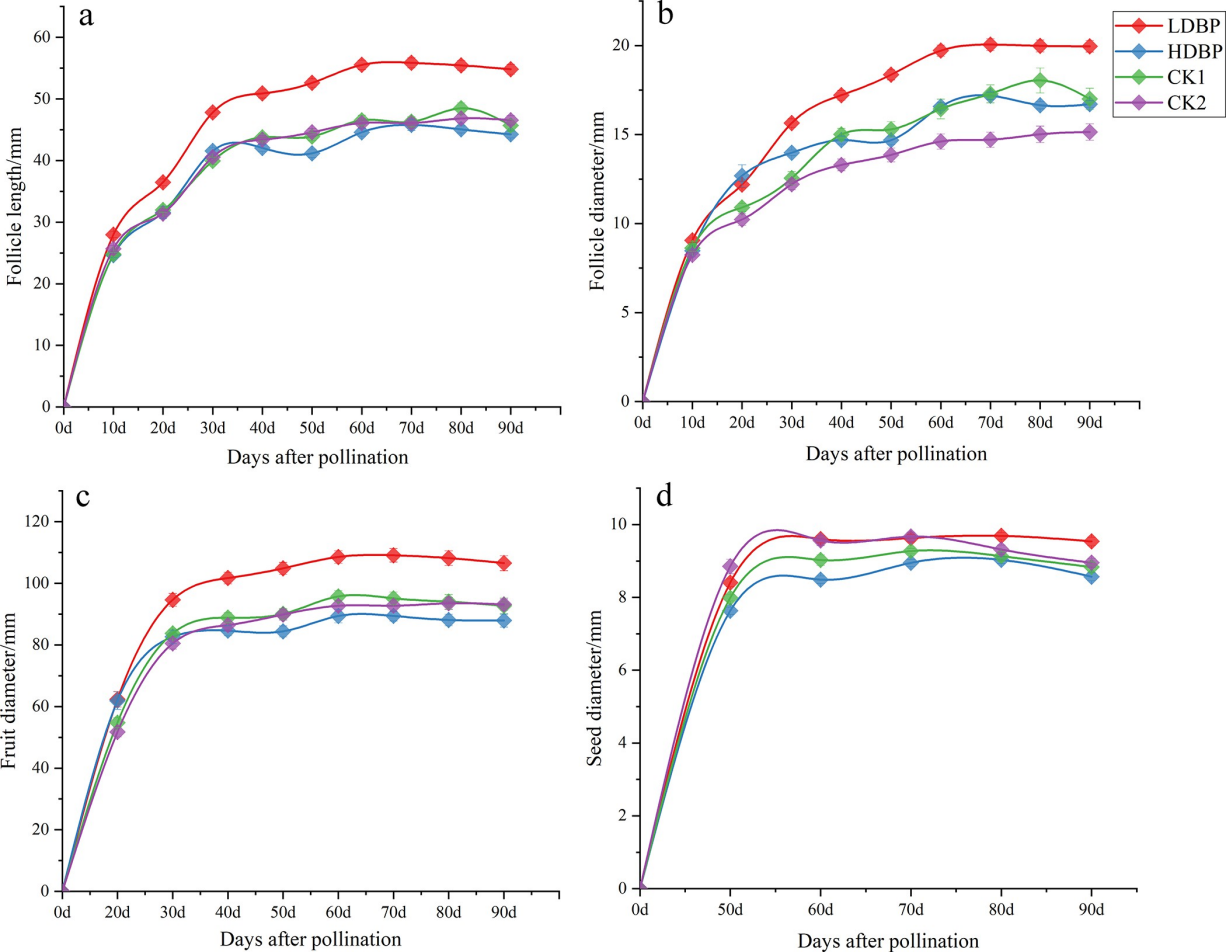
Growth and development curves of oil tree peony fruit parameters in different pollination treatments. a. the follicle length, b. the follicle diameter, c. the fruit diameter, and d. seed diameter.

According to fruit growth at different stages (**Table 1**) and fruit development curve (**Fig 3**), we found that there was no significant difference in seed growth rate. However, during the first stage of fruit development (phase Ⅰ), statistical analysis demonstrated that the accumulative increase of follicle length (19.83±0.45 mm/stage), follicle diameter (6.59±0.21mm/stage) and fruit diameter (32.28±1.67 mm/stage) were significantly larger for the LDBP group than for the HDBP, CK1 and CK2 groups (F = 13.518, p<0.01; F = 19.748, p<0.01; F = 7.149, p<0.01, respectively).

**Table 1:** Accumulative increase rate of oil tree peony fruit at different development stages.

Fruit development stage	Different treatments	Sample Size	Accumulative increase rate (mm/stage)		
			Follicle length	Follicle diameter	Fruit diameter
		(No. fruits)			
Phase Ⅰ	LDBP	52	19.83±0.45 a	6.59±0.21 a	32.28±1.67 a
	HDBP	60	16.89±0.53 b	5.51±0.16 b	20.69±1.46 b
	CK1	57	15.14±0.67 c	3.93±0.38 c	29.00±2.21 a
	CK2	57	14.88±0.71 c	3.97±0.33 c	28.74±1.95 a
Phase Ⅱ	LDBP	57	7.72±1.15 a	4.07±0.40 a	13.9±0.89 a
	HDBP	59	3.60±0.81 b	2.61±0.28 b	7.70±1.16 b
	CK1	57	6.59±1.01 a	3.89±0.60 a	11.99±1.53 a
	CK2	57	5.50±0.89 ab	2.40±0.45 b	12.22±1.49 a
Phase Ⅲ	LDBP	52	1.18±1.13 a	0.24±0.50 a	-1.99±0.92 a
	HDBP	54	-0.04±0.69 a	0.08±0.29 a	-1.47±1.26 a
	CK1	46	-1.12±1.05 a	0.16±0.65 a	-2.52±0.89 a
	CK2	46	0.24±1.05 a	0.09±0.64 a	-1.45±0.69 a

Note: Different small letter in the same column meant significant difference at 0.05 level.

In the slow fruit development period (phase Ⅱ), the accumulative increase of follicle length (7.72±1.15 mm/stage), follicle diameter (4.07±0.40 mm/stage) and fruit diameter (13.9±0.89 mm/stage) were significantly larger for the LDBP group than for the LDBP group (F = 3.267, p<0.05; F = 3.261, p<0.05; F = 3.943, p<0.05, respectively). But there were no significant difference (p>0.05) between the LDBP and controls (CK1 and CK2).

During the stable period of fruit development (phase Ⅲ), no significant differences were observed between the different treatment groups in the accumulative increase of fruit diameter, follicle length and diameter (F = 1.71, p = 0.322; F = 0.02, p = 0.996; F = 0.252, p = 0.86, respectively).

#### 3.2.2. Daily increase rate of fruit

**Fig 4:**
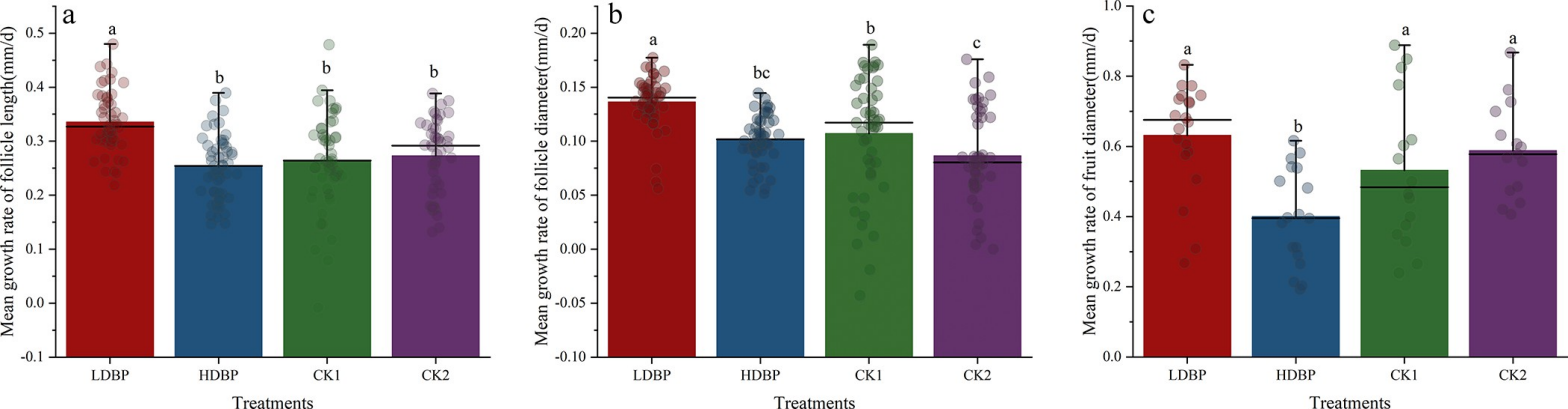
Mean fruit growth rate mm/day. a. the follicle length, b. the follicle diameter, and c. the fruit diameter. Note: Different small letter in the same column meant significant difference at 0.05 level.

The positive effect of bee pollination in the LDBP group was evident throughout the entire course of fruit development (**Fig 4**). The daily increase rate of follicle length and diameter in LDBP group were significantly high that in HDBP, CK1 and CK2 groups (F = 15.277, p<0.01; F = 15.452, p< 0.01, respectively), they had 36.00%, 30.77%, and 25.93% higher follicle length and 40.00%, 27.27% and 55.56% higher follicle diameter than that in HDBP, CK1 and CK2 groups.

Statistically, the daily increase rate of fruit diameter in LDBP group was significantly high that in HDBP, and had 18.87% higher daily increase rate (F = 7.138, p<0.01). However, there were no significant differences between the LDBP, CK1 and CK2 groups (p>0.05).

### 3.3. Fruit yield parameters

**Fig 5:**
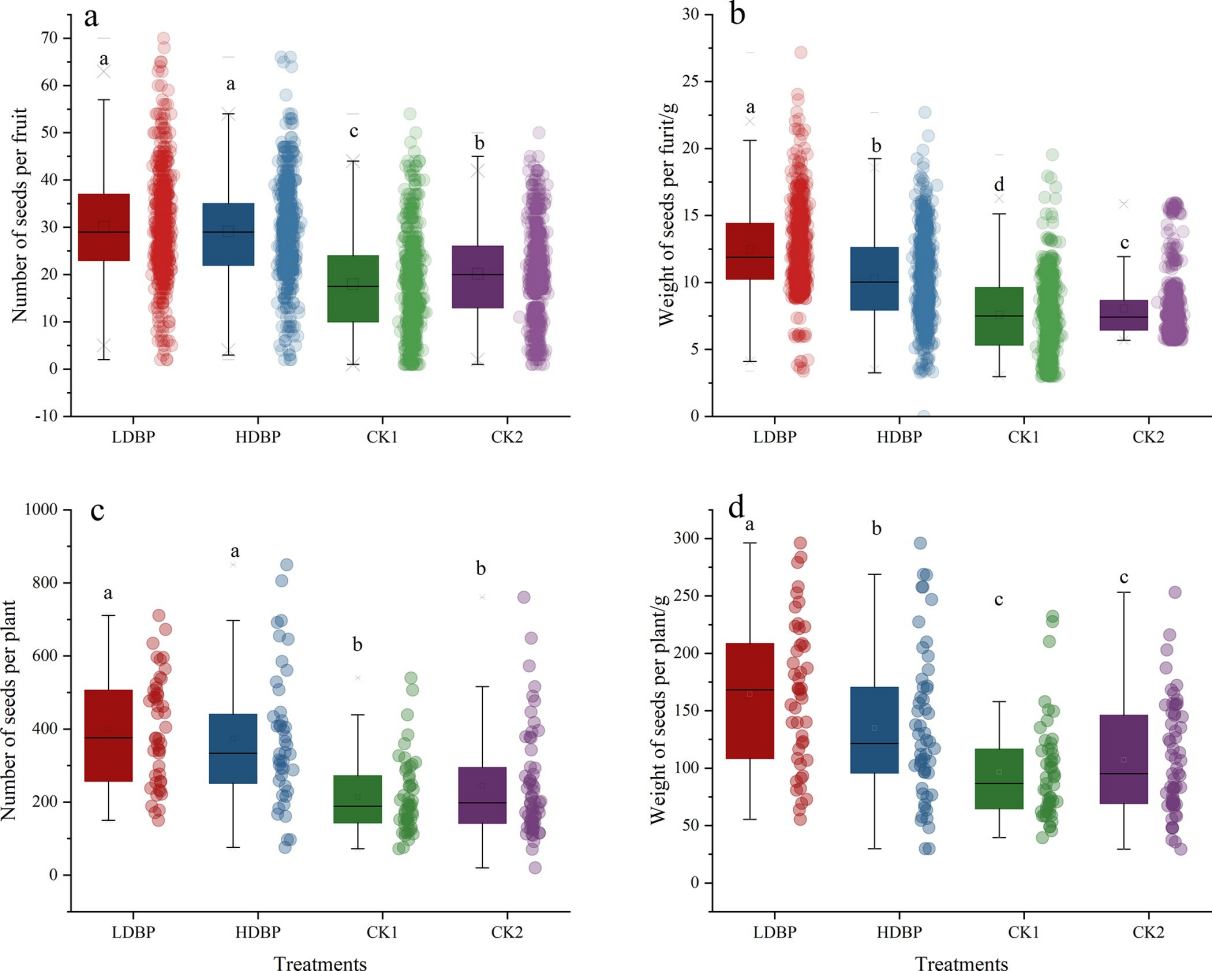
Production of oil tree peony fruits in different pollination treatments. Note: Different small letter in the same column meant significant difference at 0.05 level.

Results indicated that supplementary honey bee pollination significantly influenced oil tree peony yield parameters (**Fig 5**). Oil tree peony in LDBP group had 67.69% and 49.55% more number of seeds per fruit, 86.58% and 63.17% more number of seeds per plant than plants in CK1 and CK2 groups (F = 177.209, p<0.01; F = 19.633, p<0.01, respectively). However, oil tree peony in LDBP group had 20.72%, 64.51% and 54.71% higher weight of seeds per fruit, 21.90%, 70.26% and 53.47% higher weight of seeds per plant than HDBP, CK1 and CK2 groups (F = 288.819, p<0.01; F = 14.951, p<0.01, respectively).

### 3.4. Oil content and fatty acid content in oil

Statistically, supplementary honey bee pollination has no significant effect on seed oil quality (**Table 2**). Oil tree peony in LEDP group had 26.15% higher oil content than that in CK2 (F = 7.10, p<0.05). The kernel percent and fatty acid content in oil were not significantly different between treatments (F = 0.942, p = 0.447; F = 2.048, p = 0.166, respectively).

**Table 2:** Kernel percent, oil content and quantitative analysis of main fatty acid (%) of oil tree peony.

Pollination	Kernel percent	Oil content	Palmitic acid	Stearic acid	Oleic acid	Linoleic acid	α-linolenic acid	Unsaturated fatty acid
LDBP	58.33±3.20 a	22.19±0.48 a	5.83±0.07 b	1.48±0.06 a	22.82±0.08 b	24.18±0.04 b	42.58±0.24 a	89.58±0.17 a
HDBP	55.32±1.09 a	21.62± 0.71 a	6.06±0.03 a	1.58±0.03 a	23.32±0.16 b	25.04±0.19 a	41.42±0.12 b	89.78±0.08 a
CK1	52.69±3.01 a	20.37± 0.37 a	5.89±0.06 b	1.56±0.07 a	22.92±0.13 b	24.28±0.05 ab	42.12±0.29 ab	89.33±0.24 a
CK2	55.59± 0.52 a	17.59±1.22 b	5.81±0.08 b	1.33±0.07 b	24.63±0.47 a	21.65±0.47 c	42.92±0.45 a	89.20±0.44 a

Note: Different small letter in the same column means significant difference at 0.05 level.

## 4. Discussion

Supplementary bee pollination can significantly increase fruit growth rate and fruit yield of oil tree peony. In addition, our pollination practice experiments showed that LDBP and HDBP have better pollinating effect and leading to much faster growth and higher fruit production compared to other treatments. Bee pollination has been reported to significantly increase seed rate and yield in oil tree peony [37, 38]. Compared to non-bee-controls, long-distance and right moment outcross pollination of insects in the area with bee pollination can give crops a full opportunity for selective insemination, improve hybridization, and increase the yield and quality of fruits and seeds [9, 10]. In the oil tree peony farm, we observed that honeybees inspect and visit flowers systematically over several hours a day (8:00 a.m.-18:00 p.m.) and during the entire blooming season, covering all the space within their foraging range. They pollinated oil tree peony uniformly over space and time [37]. Thus, compared with the areas with insufficient pollination (CK2), those supplemented with bee-pollination (LDBP) have increased seed and oil content by 53.47% and 26.15%, respectively.

In our study, oil tree peony set by bee-pollinated flowers had not only a higher number of seeds per fruit, but low variability of seed quantity and more homogeneous grain size between fruits, indicating a more stable pollination service. In related studies, bee pollination significantly increased fruit set and seed set, and reduced the percentage of empty and/or deformed shells rate in pear, tea-oil trees, soybean, rapeseed and tangelos [42–46]. In addition, the oil tree peony pods pollinated by bees naturally crack and the seeds fall off naturally, relative to control groups (**Fig 2**). In addition, control groups produced few seeds, but large and heavy, as they would allocate more resources to the seeds they can set.

Moreover, our study showed that LDBP has higher pollination efficiency than HDBP. It is generally assumed that more bee densities increase pollination and crop productivity, but effects might be nonlinear [27, 47, 48]. Peace et al. (2020) reported that at realistic bee densities, the optimal orchard had 65–75% female flowers, and the most benefit was gained from the first 6–8 bees/1000 flowers, with diminishing returns thereafter [49]. Our findings are similar to this, about 60 flowers/m^2^ in our study, the pollination effect of 22 bees/m^2^ was better than 44 bees/m^2^, however, the pollination effect of lower densities needs further study. Sáez et al. (2014) reported that pollen loads on stigmas increased with visit frequency of all bees combined and particularly with visitation by *A*. *mellifera*, but drupelet set was not pollen limited along the gradient of bee abundance. However, the authors also suggest that although mainstream pollinator management relies on the assumption that more visits enhance fruit set, high bee visitation rates can be detrimental for fruit development and, consequently, for crop yield [50]. The relationship between bee visiting frequency and stigma pollen deposition on oil tree peony needs further analysis. In addition, temperature also significantly affects bees’ flower-visiting activities on plants [37, 38, 51]. During the test, the indoor temperature of the net was higher than the outdoor temperature of the net, and when the temperature was high at noon, a large number of bees hit the top. Therefore, we guessed that the net chamber environment restricted the activities of higher-density bee colonies, affecting their flower-visiting activities on oil tree peony, thereby affecting the yield.

Throughout the developmental period, the growth rate of oil tree peony fruit in LDBP was significantly higher than those of HDBP or controls (CK1, CK2). The growth rate of fruit is closely related to early fertilization. More pollen deposition can cause early development of ovary and ovule [52–54]. In oilseed rape, studies have reported that bee pollination increased unsaturated fatty acid levels by 0.3%, increased chlorophyll and seed oil content, and shortened rapeseed maturity [16, 42, 55]. More, bee pollination increases sunflower weight by 91%, vitamin E levels by 45% and unsaturated fatty acid levels by 0.3% [56]. In the current study, bee pollination most prominently enhanced the seed quantity, leading to heavier seeds, increased oil content, and decreased seed deformity rate. But it had no significant effect on the content of UFA in seed oil, which was the same as the results of He et al. (2020) [38]. Whether other oil quality parameters and seed nutritional values are similarly affected by the pollination mode demands further investigation for crops and quality measures.

Although the scale of our study is limited to just on control pollination net room and one season, our research found that honeybees (*A*. *mellifera*) provide valuable pollination services for oil tree peony. This proves the pollination potential of honeybees in farms and their strong adaptability to crops [57, 58]. However, the pollination practice of honeybees in large-scale oil tree peony farms needs to be further studied to explore the potential effects of management of honeybees and wild pollinators in oil tree peony pollination. Our analyses suggest that increase conservation strategies for managed bees and wild insect provide pollination services for the development of modern, intensive agriculture to minimize the risk of pollination deficits affecting food production.

## Supporting information

S1 Data (XLSX)
